# Mental Health Disorders and Coping Strategies Among Palestinian Refugees in Egypt During the 2023 War on Gaza: A Cross-Sectional Study

**DOI:** 10.1177/00207640251414176

**Published:** 2026-01-31

**Authors:** Noha Fadl, Ammar Elsayed Mohamed Mohamed shahtou, Hagar Mostafa Own, Muhammad Abdullatif Alkasaby, Suzan Abdel-Rahman, Rola Mahmoud Abdallah Tafesh, Saja Hasan Alzaanin, Hisham Mohammed Mahmoud Zourob, Mohammed Walid Ali Aljedaili, Fathi I. A. Shaheen, Rofaida Gamal Abdullah

**Affiliations:** 1 Family Health Department, High Institute of Public Health, Alexandria University, Alexandria, Egypt; 2 ERADAH Mental Health Complex, Najran, Saudi Arabia; 3 Abbassia Psychiatric Hospital, Cairo, Egypt; 4 Health in Humanitarian Crises Centre, London School of Hygiene & Tropical Medicine, London, UK; 5 Biostatistics and Demography, Faculty of Graduate Studies for Statistical Research, Cairo University, Egypt; 6 Faculty of Medicine, Mansoura University, Mansoura, Egypt; 7 Faculty of Dentistry, Al-Azhar University, Gaza, Palestine; 8 Faculty of Medicine, Zagazig University, Zagazig, Egypt; 9 Faculty of Medicine, Suez Canal University, Ismailia, Egypt; 10 Faculty of Mary Lou Fulton, Arizona State University, Arizona, USA

**Keywords:** PTSD, anxiety, depression, psychological distress, coping, Gaza

## Abstract

**Background:**

The ongoing war on Gaza has a devastating impact on Palestinians, particularly on their psychological well-being.

**Aims:**

To assess mental health disorders and coping strategies among Palestinians displaced to Egypt during the 2023 Gaza war.

**Methods:**

A cross-sectional study included Palestinian refugees older than 18 years. Impact of Event Scale-6, Generalized Anxiety Disorder-7, Patient Health Questionnaire-9, and Coping Strategies Inventory-Short Form were used to assess posttraumatic stress disorder (PTSD), anxiety, depression, and coping mechanisms, respectively.

**Results:**

Among the 558 participants, 62.2% were females, with a mean age of 33.91 ± 11.84 years. Prevalence rates of PTSD, anxiety, and depression were 37.5%, 94.1%, and 94.8%, respectively. Emotion disengagement was the most adopted coping strategy. Multiple linear regression showed that PTSD was positively associated with being female, anxiety, depression, and the use of problem engagement, problem disengagement, and emotion disengagement coping strategies. Very good financial status and difficulty accessing healthcare were negatively associated with PTSD. Anxiety was associated with older age, being female, difficulty finding a job, war-related injury, PTSD, depression, and problem disengagement, while emotion engagement was protective. Depression was associated with a family history of mental illness, housing difficulties, emotion disengagement, anxiety, and PTSD, while problem engagement and being female were protective.

**Conclusions:**

Context-sensitive psychosocial interventions that strengthen coping strategies are essential to alleviate the psychological burden among this vulnerable population.

## Introduction

The United Nations High Commissioner for Refugees (UNHCR) defines refugees as individuals forced to leave their country because of persecution, war, or violence (UNHCR, 2024a). Notably, the Eastern Mediterranean Region (EMR) is the epicenter of the world’s most violent conflicts and hosts the largest global refugee population (Stein et al., 2021).

By the end of 2024, 123.2 million people worldwide were forcibly displaced, including 73.5 million internally displaced persons (IDPs) and 42.7 million refugees (UNHCR, 2024b). In the EMR, over 14.3 million Sudanese were displaced, including 11.5 million IDPs and 2.8 million refugees. Further, there were 6.1 million Syrian refugees and 7.4 million internally displaced within the country, while 4.8 million were internally displaced in Yemen. Further, the ongoing war in Gaza has uprooted up to 2 million—90% of the population—to flee within the Gaza Strip, with some having been forced to flee multiple times (UNHCR, 2024b).

Exposure to conflict-related trauma and forced displacement exposes individuals to a heightened risk of mental health disorders, including post-traumatic stress disorder (PTSD), anxiety, and depression. This vulnerability arises from multiple traumatic experiences, such as the loss of family members, physical injury, destruction of homes, and prolonged uncertainty regarding basic needs, including food, shelter, and healthcare (Ahmed et al., 2024; Ojha et al., 2024).

A systematic review indicates that prevalence rates of PTSD, anxiety, and depression among Syrian conflict-affected refugees were 26% to 45%, 30% to 32%, and 40% to 45%, respectively (Ojha et al., 2024). Another systematic review revealed that refugees resettling in high-income countries experienced notable rates of mental health disorders, with diagnosed and self-reported cases of post-traumatic stress disorder (PTSD) at 29% and 37%, respectively; depression at 30% and 40%; and anxiety at 13% and 42%. (Henkelmann et al., 2020).

Coping strategies include both cognitive and behavioral strategies adopted by individuals to manage the stressors they face throughout life (Addison et al., 2024). Coping strategies are generally categorized into engagement strategies, which confront and manage negative experiences to support psychological well-being, and disengagement strategies, which involve avoiding such experiences (Bourguignon et al., 2020). Coping strategies can also be divided into problem-focused and emotion-focused approaches. Problem-focused coping entails actions aimed at changing or removing stressful situations, whereas emotion-focused coping centers on managing emotional reactions, such as by reinterpreting the event or avoiding it altogether (Schoenmakers et al., 2015).

Refugees employ a range of coping strategies to manage stress and trauma. These strategies can be religious activities, seeking social and emotional support, thinking positively about the future, reframing situations in a constructive way, and using emotion-focused techniques. Additionally, practices like avoidance, memory suppression, and distraction are frequently observed (Figueiredo & Petravičiūtė, 2025).

Egypt is among the largest host countries in the EMR, receiving over 100,000 people fleeing Gaza between October 2023 and March 2024 (United Nations Office for the Coordination of Humanitarian Affairs [OCHA], 2024). Despite extensive research on refugee mental health, gaps remain regarding Palestinians who migrated to Egypt, particularly in integrating coping strategies with mental health outcomes. Accordingly, this study aimed to assess mental health disorders and coping strategies among Palestinian refugees in Egypt during the 2023 to 2024 Gaza war, contributing to efforts to integrate mental health care into universal health coverage and advance Sustainable Development Goal 3: ensuring healthy lives and promoting well-being for all (BOCCO & FROEHLICH, 2022).

## Methods

### Study Design and Study Setting

This cross-sectional study included Palestinians older than 18 years and displaced to Egypt during the 2023 Gaza war. Data was collected between February 1 and April 15, 2025, using anonymous online surveys distributed through various social media channels (e.g., WhatsApp, Facebook, X, and Telegram) in addition to face-to-face interviews. Participants with incomplete data were excluded.

### Sample Size and Sampling Technique

Using an assumed prevalence rate of 50% for PTSD among refugees, a 5% accepted degree of precision, an α of 0.05, and a power of 95%, the minimum required sample size was 384 responses using Cochran’s formula. We successfully collected 558 valid responses, which enhanced the study’s statistical power. Given the challenges in accessing a random sample of Palestinian refugees in Egypt, a combination of convenience and snowball sampling methods was employed to recruit participants.

### Measures

The outcomes of interest were PTSD, anxiety, and depression. Symptoms of PTSD were assessed using the Arabic version of the **Impact of Events Scale-6** (IES-6) (Hemade et al., 2025). It consists of six items, with a suggested cut-off point of 15 as the ideal cut-off point to screen for PTSD (Abas et al., 2023). Participants were asked to score how much they were distressed or bothered in the past 7 days with respect to events related to the 2023 Gaza war. Each item is rated on a five-point scale ranging from 0 “not at all” to 4 “extremely” (Abas et al., 2023). In the present study, the IES-6 showed a Cronbach’s alpha of 0.701.

The Arabic version of **General Anxiety Disorder-7** (GAD-7) was used to assess the severity of generalized anxiety disorder (Spitzer et al., 2006). Participants were asked how many times they had been bothered by each symptom over the past 2 weeks, with response choices of not at all, several days, more than half the days, and nearly every day, recorded as 0, 1, 2, and 3, respectively. GAD-7 scores were categorized as 0 to 4 for none/minimal, 5 to 9 for mild, 10 to 14 for moderate, and 15 to 21 for severe anxiety (Spitzer et al., 2006). Cronbach’s alpha value of the GAD-7 was 0.896.

The Arabic version of the **Patient Health Questionnaire-9** (PHQ-9) was used to assess the severity of depression (Spitzer et al., 1999). Participants were asked how many times they had been bothered by each symptom over the past 2 weeks, with response choices of not at all, several days, more than half the days, and nearly every day, scored as 0, 1, 2, and 3, respectively. Participants were classified into four groups: none (0–4), mild (5–9), moderate (10–14), moderately severe (15–19), and severe (20–27) depression (Spitzer et al., 1999). Cronbach’s alpha for the PHQ-9 was 0.875.

The independent variables included the sociodemographic characteristics of the participants, such as age, gender, educational level, and financial status. The presence of chronic diseases and family history of mental illness was also collected. Data about post-migration challenges was added, such as difficulties in finding appropriate housing, obtaining a suitable job, accessing healthcare and medications, attending educational institutions, worrying about running out of food, struggles with social integration, and experiences of discrimination. Exposure to war-related events was included (e.g., suffering a physical injury and death of a family member) (Supplemental File 1).

The Arabic version of the **Coping Strategies Inventory Short-Form** (CSI-SF) (Awad et al., 2022) was used to measure four coping strategies based on 16 items: problem-focused engagement, problem-focused disengagement, emotion-focused engagement, and emotion-focused disengagement. Participants were instructed to assess how often they use each coping strategy as follows: 1 = “Never,” 2 = “Seldom,” 3 = “Sometimes,” 4 = “Often” and 5 = “Almost Always.” Higher scores indicated greater use of the selected coping strategy (Addison et al., 2007). The Cronbach’s alpha for the CSI-SF was 0.720.

### Statistical Analysis

Descriptive analysis was conducted to summarize the characteristics of the study participants. Differences in outcome scores (e.g., PTSD, anxiety, and depression) were analyzed using independent sample t-tests and one-way ANOVA. The t-test compared mean scores between two unrelated groups, while ANOVA was used for comparisons among three or more independent groups. Effect sizes were calculated for t-tests and one-way ANOVA to measure the magnitude of these associations. We calculated Cohen’s d for t-tests (0.2: small effect, 0.5: medium effect, 0.8: large effect) and Eta-squared (η²) for one-way ANOVA (0.01: small effect, 0.06: medium effect, 0.14: large effect) (Lakens, 2013). Pearson’s correlation coefficient (r) was used to measure the strength and direction of association between two quantitative variables.

Multiple linear regression models were performed to identify the key factors influencing PTSD, anxiety, and depression. Three separate regression models were constructed for each outcome variable. The variance inflation factor (VIF) was checked to exclude multicollinearity. The adjusted R² was calculated to assess the extent to which the independent variables explained the variance in data. The adjusted R² was 0.43 for the PTSD model, 0.67 for the anxiety model, and 0.62 for the depression model. Additionally, an ANOVA test was conducted to verify the overall significance of the models. The results were statistically significant (*p* < .001) in all models, demonstrating the goodness-of-fit of the models and their ability to explain the outcomes studied. Cronbach’s alpha was used to measure the internal consistency and reliability of the IES-6, GAD-7, PHQ-9, and CSI-SF. Values of Cronbach’s alpha coefficients ⩾0.7 indicate adequate internal consistency (Taber, 2018). A two-tailed *p* value < 0.05 was considered statistically significant. Statistical testing was performed using the Statistical Package for Social Sciences (SPSS), version 28.0 (IBM Corp., Armonk, NY, USA).

### Ethical Considerations

Ethical approval was obtained from the High Institute of Public Health, Alexandria University, Egypt (IRB number 00013692). The Declaration of Helsinki was followed throughout the study. For the face-to-face interviews, informed consent was obtained from the participants prior to completing the survey. For the online survey, a question of voluntary participation was required to access the survey.

## Results

Across the 558 participants, 62.2% were females, and 62.5% were university graduates or higher, with a mean age of 33.91 ± 11.84 years. Around 39% of the participants considered their financial status to be poor or very poor. Nearly one-third reported physical injuries (34.2%), and 28.3% lost a family member due to the war. The most reported stressors after displacement to Egypt were getting a suitable job (85.1%), followed by finding appropriate housing (76.5%) and accessing educational institutions (71.9%). Prevalence rates of PTSD, anxiety, and depression were 37.5%, 94.1%, and 94.8%, respectively. Other characteristics of study participants are presented in Table 1. Specific responses of the participants to IES-6, GAD-7, and PHQ-9 are described in Supplemental File 2.

**Table 1.** Characteristics of the Study Participants (*n* = 558).

Characteristics		*n* (%)
Responses	Face-to-face online	147 (26.3) 411 (73.7)
Age, years	(mean ± SD)	33.91 ± 11.84
Gender	Male	211 (37.8)
	Female	347 (62.2)
Education level	Less than high school	43 (7.7)
	High school	166 (29.7)
	University or higher	349 (62.5)
Financial status	Very poor	70 (12.5)
	Poor	150 (26.9)
	Fair	184 (33.0)
	Good	103 (18.5)
	Very good	51 (9.1)
Presence of chronic disease	Yes	125 (22.4)
	No	433 (77.6)
Family history of mental illness	Yes	89 (15.9)
	No	469 (84.1)
Post-migration stressors: finding appropriate housing	Yes	427 (76.5)
	No	131(23.5)
Post-migration stressors: obtaining suitable job	Yes	475 (85.1)
	No	83 (14.9)
Post-migration stressors: accessing healthcare and medications	Yes	382 (68.5)
	No	176(31.5)
Post-migration stressors: attending educational institutions	Yes	401 (71.9)
	No	157 (28.1)
Post-migration stressors: worry about running out of food	Yes	271 (48.6)
	No	287 (51.4)
Post-migration stressors: struggles with social integration	Yes	326 (58.4)
	No	232 (41.6)
Post-migration stressors: experiences of discrimination	Yes	172 (30.8)
	No	386 (69.2)
War-related trauma: suffering a physical injury	Yes	191 (34.2)
	No	367 (65.8)
War-related trauma: death of a family member	Yes	158 (28.3)
	No	400 (71.7)
PTSD symptoms	Yes	349 (62.5)
	No	209 (37.5)
Anxiety symptoms	None–minimal	33 (5.9)
	Mild	104 (18.6)
	Moderate	146 (26.2)
	Severe	275 (49.3)
Depression symptoms	None–minimal	29 (5.2)
	Mild	106 (19.0)
	Moderate	131 (23.5)
	Moderately severe	148 (26.5)
	Severe	144 (25.8)

Among the coping strategies reported by participants, emotion disengagement was the most frequently used (13.25 ± 3.23), followed by problem disengagement (13.18 ± 2.98) and problem engagement (12.04 ± 2.81), and the least utilized was emotion engagement strategies (11.32 ± 3.52) (Table 2).

**Table 2.** Coping Mechanisms Adopted by the Participants.

Items	Mean ± SD
Emotion engagement	11.32 ± 3.52
I try to let my emotions out.	2.84 ± 1.11
I try to talk about it with a friend or family.	2.71 ± 1.19
I let my feelings out to reduce the stress.	2.98 ± 1.11
I ask a close friend or relative that I respect for help or advice.	2.78 ± 1.15
Problem engagement	12.04 ± 2.81
I make a plan of action and follow it.	2.55 ± 1.03
I look on the bright side of things.	3.15 ± 1.04
I tackle the problem head on.	3.21 ± 1.09
I step back from the situation and try to put things into perspective.	3.14 ± 1.04
Emotion disengagement	13.25 ± 3.23
I try to spend time alone.	3.57 ± 1.12
I tend to blame myself.	3.07 ± 1.21
I tend to criticize myself.	3.04 ± 1.21
I keep my thoughts and feelings to myself.	3.57 ± 1.14
Problem disengagement	13.18 ± 2.98
I hope the problem will take care of itself.	3.50 ± 1.14
I try to put the problem out of my mind.	2.76 ± 1.09
I hope for a miracle.	4.05 ± 1.19
I try not to think about the problem.	2.87 ± 1.15

Bivariate analysis showed that PTSD symptoms were significantly higher among participants aged 30 to 44 years, females, university graduates or higher, those with very poor financial status, family history of mental illness, difficulties in finding appropriate housing and suitable jobs, concerns about food insecurity, barriers to social integration, those who experienced physical injuries or lost a family member due to the war, and those who reported anxiety and depression symptoms (*p* < .05). The effect sizes for all significant variables ranged from small to medium, except anxiety and depression symptoms demonstrated a large effect size (η² = 0.321 and 0.254, respectively) (Table 3).

**Table 3.** Characteristics of the Study Participants by Post-Traumatic Stress Disorder.

Characteristics		Mean ± SD	*p* Value	Effect size
Age, years	18-29	15.86 ± 4.90	0.006*	0.02^b^
	30-44	17.8 ± 3.62		
	⩾45	16.99 ± 3.98		
Gender	Male	15.16 ± 5.09	<0.001*	0.512^a^
	Female	17.32 ± 3.60		
Education level	Less than high school	14.44 ± 6.15	0.004*	0.021^b^
	High school	16.50 ± 4.49		
	University or higher	16.76 ± 3.95		
Financial status	Very poor	18.37 ± 3.81	<0.001*	0.06^b^
	Poor	16.86 ± 3.96		
	Fair	16.63 ± 4.01		
	Good	15.78 ± 4.49		
	Very good	13.90 ± 5.55		
Presence of chronic disease	Yes	16.76 ± 4.11	0.22	0.075^a^
	No	16.43 ± 4.42		
Family history of mental illness	Yes	17.35 ± 4.81	0.03*	0.23^a^
	No	16.34 ± 4.25		
Post-migration stressors: finding appropriate housing	Yes	16.80 ± 4.40	0.003*	0.05^a^
	No	15.54 ± 4.06		
Post-migration stressors: obtaining suitable job	Yes	16.73 ± 4.24	0.007*	0.35^a^
	No	15.20 ± 4.77		
Post-migration stressors: accessing healthcare and medications	Yes	16.54 ± 4.53	0.766	0.02^a^
	No	16.43 ± 3.95		
Post-migration stressors: attending educational institutions	Yes	16.63 ± 3.98	0.250	0.10^a^
	No	16.18 ± 4.48		
Post-migration stressors: worry about running out of food	Yes	16.89 ± 4.76	0.043*	0.17^a^
	No	16.14 ± 3.90		
Post-migration stressors: struggles with social integration	Yes	16.84 ± 4.44	0.03*	0.18^a^
	No	16.03 ± 4.20		
Post-migration stressors: experiences of discrimination	Yes	16.50 ± 5.07	0.491	0.002^a^
	No	16.51 ± 399		
War-related trauma: suffering a physical injury	Yes	17.13 ± 4.18	0.014*	0.22^a^
	No	16.18 ± 4.41		
War-related trauma: death of a family member	Yes	17.33 ± 3.98	0.002*	0.267^a^
	No	16.18 ± 4.45		
Anxiety symptoms	None- minimal	10.63 ± 4.70	<0.001*	0.321^b^
	Mild	13.40 ± 4.45		
	Moderate	16.07 ± 3.14		
	Severe	18.61 ± 3.29		
Depression symptoms	None-minimal	11.79 ± 4.28	<0.001*	0.254^b^
	Mild	13.68 ± 4.65		
	Moderate	15.84 ± 4.02		
	Moderately severe	17.49 ± 3.11		
	Severe	19.13 ± 3.29		

Effect size: ^a^Cohen’s d, ^b^Eta-squared; *p < .05.

Tables 4 and **5** indicated that symptoms of anxiety and depression were significantly higher among individuals aged 30 to 44, females, those with a university degree or higher education, individuals reporting very poor financial status, those with chronic illnesses, and individuals with a family history of mental illness. Elevated symptoms were also associated with all post-migration stressors and all war-related experiences (*p* < .05). The effect sizes for these significant variables ranged from small to medium. Anxiety was strongly associated with both PTSD and depression, with large effect sizes. Similarly, depression showed a significant association with both PTSD and anxiety, also with large effect sizes.

**Table 4.** Characteristics of the Study Participants by Anxiety Symptoms.

Characteristics		Mean ± SD	*p* Value	Effect size
Age, years	18-29	11.97 ± 5.67	<0.001*	0.09^b^
	30-44	15.52 ± 4.59		
	⩾45	14.71 ± 5.47		
Gender	Male	11.94 ± 5.71	<0.001*	0.54^a^
	Female	14.83 ± 5.12		
Education level	Less than high school	12.16 ± 6.23	0.056	0.10^b^
	High school	13.37 ± 6.03		
	University or higher	14.11 ± 5.14		
Financial status	Very poor	16.51 ± 4.66	<0.001*	0.07^b^
	Poor	14.82 ± 4.93		
	Fair	13.29 ± 5.47		
	Good	12.48 ± 5.49		
	Very good	10.88 ± 6.31		
Presence of chronic disease	Yes	15.47 ± 5.07	<0.001*	0.41^a^
	No	13.23 ± 5.56		
Family history of mental illness	Yes	16.09 ± 5.16	<0.001*	0.52^a^
	No	13.29 ± 5.49		
Post-migration stressors: finding appropriate housing	Yes	14.418 ± 5.17	<0.001*	0.53^a^
	No	11.54 ± 6.08		
Post-migration stressors: obtaining suitable job	Yes	14.28 ± 5.20	<0.001*	0.68^a^
	No	10.63 ± 6.29		
Post-migration stressors: accessing healthcare and medications	Yes	14.36 ± 5.32	<0.001*	0.36^a^
	No	12.38 ± 5.74		
Post-migration stressors: attending educational institutions	Yes	14.09 ± 5.45	0.008*	0.23^a^
	No	12.82 ± 5.63		
Post-migration stressors: worry about running out of food	Yes	14.67 ± 5.34	<0.001*	0.33^a^
	No	12.86 ± 5.57		
Post-migration stressors: struggles with social integration	Yes	14.49 ± 5.31	<0.001*	0.33^a^
	No	12.68 ± 5.65		
Post-migration stressors: experiences of discrimination	Yes	14.63 ± 5.26	0.009*	0.24^a^
	No	13.33 ± 5.60		
War-related trauma: suffering a physical injury	Yes	15.23 ± 5.38	<0.001*	0.42^a^
	No	12.96 ± 5.45		
War-related trauma: death of a family member	Yes	15.37 ± 4.94	<0.001*	0.42^a^
	No	13.09 ± 5.62		
PTSD symptoms	Yes	15.97 ± 4.44	<0.001*	1.27^a^
	No	10.00 ± 5.13		
Depression symptoms	None-minimal	3.55 ± 3.72	<0.001*	0.57^b^
	Mild	8.89 ± 3.46		
	Moderate	12.25 ± 3.97		
	Moderately severe	15.87 ± 3.59		
	Severe	18.51 ± 3.40		

Effect size: ^a^Cohen’s d, ^b^Eta-squared ;*p < .05.

**Table 5.** Characteristics of the Study Participants by Depression Symptoms.

Characteristics		Mean ± SD	*p* Value	Effect size
Age, years	18-29	13.66 ± 6.42	<0.001*	0.03^b^
	30-44	16.23 ± 6.14		
	⩾45	15.53 ± 6.71		
Gender	Male	13.71 ± 6.46	<0.001*	0.3^a^
	Female	15.64 ± 6.39		
Education level	Less than high school	12.88 ± 6.77	0.1	0.01^b^
	High school	15.07 ± 6.81		
	University or higher	15.09 ± 6.25		
Financial status	Very poor	18.48 ± 5.98	<0.001*	0.05^b^
	Poor	15.75 ± 5.88		
	Fair	14.46 ± 6.37		
	Good	13.36 ± 6.36		
	Very good	12.29 ± 7.20		
Presence of chronic disease	Yes	16.90 ± 6.43	<0.001*	0.40^a^
	No	14.34 ± 6.39		
Family history of mental illness	Yes	18.31 ± 6.58	<0.001*	0.64^a^
	No	14.27 ± 6.26		
Post-migration stressors: finding appropriate housing	Yes	15.78 ± 6.15	<0.001*	0.59^a^
	No	12.07 ± 6.74		
Post-migration stressors: obtaining suitable job	Yes	15.39 ± 6.23	<0.001*	0.51^a^
	No	12.15 ± 7.21		
Post-migration stressors: accessing healthcare and medications	Yes	15.61 ± 6.32	<0.001*	0.34^a^
	No	13.41 ± 6.59		
Post-migration stressors: attending educational institutions	Yes	15.39 ± 6.22	0.004*	0.26^a^
	No	13.69 ± 6.99		
Post-migration stressors: worry about running out of food	Yes	16.11 ± 6.47	<0.001*	0.36^a^
	No	13.78 ± 6.31		
Post-migration stressors: struggles with social integration	Yes	15.65 ± 6.38	<0.001*	0.28^a^
	No	13.87 ± 6.49		
Post-migration stressors: experiences of discrimination	Yes	15.94 ± 6.04	0.005*	0.23^a^
	No	14.45 ± 6.63		
War-related trauma: suffering a physical injury	Yes	16.46 ± 6.35	<0.001*	0.37^a^
	No	14.11 ± 6.41		
War-related trauma: death of a family member	Yes	17.08 ± 5.94	<0.001*	0.47^a^
	No	14.06 ± 6.49		
PTSD symptoms	Yes	17.13 ± 5.83	<0.001*	1.01^a^
	No	11.22 ± 5.82		
Anxiety symptoms	None-minimal	4.46 ± 3.69	<0.001*	0.47^b^
	Mild	9.80 ± 4.58		
	Moderate	13.28 ± 4.70		
	Severe	18.95 ± 4.85		

Effect size: ^a^Cohen’s d, ^b^Eta-squared; *p < .05.

Symptoms of PTSD showed a positive correlation with coping strategies involving problem engagement (r = 0.119), problem disengagement (r = 0.385), and emotion disengagement (r = 0.431). Both anxiety and depression were positively associated with problem disengagement (r = 0.385 for anxiety and r = 0.310 for depression), as well as emotional disengagement strategies (r = 0.477 for anxiety and r = 0.505 for depression) (Table 6).

**Table 6.** Correlation Between Coping Strategies, Post-Traumatic Stress Disorder, Anxiety, and Depression.

Domains of Coping Strategies Inventory-short form	PTSD	Anxiety	Depression
Emotion engagement	0.081	−0.013	−0.59
Problem engagement	0.119*	0.003	−0.072
Emotion disengagement	0.431*	0.477*	0.505*
Problem disengagement	0.385*	0.385*	0.310*

* *p* < .05.

The multiple linear regression analysis revealed that being female (β = 0.847, *p* = .006), the use of problem engagement (β = 0.136, *p* = .018), problem disengagement (β = 0.142, *p* = .017), emotion disengagement (β = 0.160, *p* = .004), coping strategies, anxiety (β = 0.301, *p* < .001), and depression (β = 0.087, *p* = .015) were associated with higher levels of PTSD. Conversely, participants having a “very good” financial status (β = −2.201, *p* = .001) and experiencing difficulties in accessing healthcare services (β = −0.758, *p* = .021) were less likely to have PTSD (Table 7). Older age (β = 0.057, *p* < .001), being a female (β = 1.039, *p* < .001), facing difficulty in obtaining a suitable job (β = 1.355, *p* < .001), experiencing war-related physical injury (β = 0.749, *p* = .02), using a problem disengagement coping strategy (β = 0.225, *p* < .001), the presence of PTSD (β = 0.277, *p* < .001), and depression (β = 0.436, *p* < .001) increased the likelihood of anxiety. Meanwhile, the emotion engagement strategy (β = −2.201, *p* = .018) was protective against anxiety (Table 7). The likelihood of depression increased among participants with a family history of mental illness (β = 1.228, *p* = .013), experiencing challenges in finding appropriate housing (β = 1.222, *p* = .006), using an emotion disengagement coping strategy (β = 0.370, *p* < .001), reporting symptoms of anxiety (β = 0.692, *p* < .001), and PTSD (β = 0.127, *p* = .015). On the contrary, problem engagement strategy (β = −0.208, *p* = .003) and being female (β = −0.922, *p* = .014) were negatively associated with depression (Table 7).

**Table 7.** Factors Influencing PTSD, Anxiety, and Depression Using Multiple Linear Regression Models.

Predictors	PTSD (model 1)		Anxiety (model 2)		Depression (model 3)	
	B	*p* Value	β	*p* Value	B	*p* Value
Age	−.012	.396	.057	<0.001*	−.030	.085
Gender: female	.847	.006*	1.039	<0.001*	−.922	.014*
Education level: high School	.867	.141	−.368	.515	1.360	.056
Education level: university or higher	.985	.085	.078	.887	.979	.157
Financial status: poor	−.870	.078	.294	.535	−.641	.282
Financial status: fair	−.423	.397	−.287	.550	−.736	.222
Financial status: good	−.734	.190	−.230	.668	−.643	.342
Financial status: very good	−2.201	.001*	−.713	.268	−.609	.453
Presence of chronic diseases	−.458	.240	.088	.813	.772	.101
Family history of mental illness	−.378	.356	.278	.479	1.228	.013*
Post-migration stressors: finding appropriate housing	.000	.999	−.060	.865	1.222	.006*
Post-migration stressors: obtaining suitable job	−.117	.784	1.355	.001*	−.223	.667
Post-migration stressors: accessing healthcare and medications	−.758	.021*	.252	.426	.417	.295
Post-migration stressors: attending educational institutions	.235	.479	.020	.949	.134	.737
Post-migration stressors: worry about running out of food	.066	.833	−.100	.739	.153	.687
Post-migration stressors: struggles with social integration	.213	.483	.349	.232	.016	.966
Post-migration stressors: experiences of discrimination	−.542	.114	.568	.084	−.169	.684
War-related trauma: suffering a physical injury	.136	.685	.749	.020*	−.013	.975
War-related trauma: death of a family member	.049	.891	−.485	.156	.716	.096
Emotion engagement coping strategy	.046	.329	−.107	.018*	−.030	.592
Problem engagement coping strategy	.136	.018*	−.010	.855	−.208	.003*
Emotion disengagement coping strategy	.160	.004*	.065	.221	.370	<0.001*
Problem disengagement coping strategy	.142	.017*	.225	<0.001*	−.024	.744
Anxiety	.301	<0.001*			.692	<0.001*
Depression	.087	.015	.436	<0.001*		
PTSD			.277	<0.001*	.127	.015*

* *p* < .05 β: Coefficients.

## Discussion

To the best of our knowledge, this is the first study to emphasize the psychological burden of war and displacement on Palestinians to Egypt following the 2023 Gaza war. Discrepancies in prevalence rates of mental health disorders among refugees were reported. Compared to our findings, a lower prevalence rate for PTSD among Palestinian refugees residing in Jordan after the 2023 Gaza war was reported (49.2%) (Gammoh et al., 2025). Further, 43% of Iraqi refugees resettling in Turkey (Tekin et al., 2016), 29.9% of Syrian refugees in Sweden (Tinghög et al., 2017), and 15.9% of Ukrainian refugees in Denmark reported symptoms of PTSD (Karstoft et al., 2024). Further, a systematic review including 4,639 refugees reported a prevalence rate of 31.46% of PTSD (Blackmore et al., 2020). During the armed conflict in Sudan, Khalil et al. (2024) found that 36.6% of Sudanese had PTSD, with refugees having a 1.4 times higher risk of PTSD than internally displaced and non-displaced individuals. The latter study emphasized the unique stressors the refugees face, which can intensely affect their mental health. A longitudinal study between 2006 and 2021 in Palestine found that 100% of the participants were exposed to war-related traumatic experiences in 2021 (Altawil et al., 2023). While our study focused on PTSD, a globally recognized mental disorder, we acknowledge that this diagnosis might not fit Palestinians who have been experiencing continuous and intergenerational trauma under the Israeli occupation.

In alignment with our findings, Albelbeisi et al. (2025) reported alarming rates of depression and anxiety among internally displaced Palestinians between 15 June and 15 August 2024 (99.5% and 99.7%, respectively). Another study during the 2023 Gaza war found that 73% of Palestinian women residing in camps in Jordan exhibited symptoms of depression, while 60% showed signs of anxiety (Gammoh et al., 2024). Among Ukrainian war refugees, a study found a prevalence rate of 41% and 23% for depression and anxiety, respectively (Guerrero et al., 2023). Further, 46.2% and 39.6% of Sudanese refugees in Ethiopia reported depression and anxiety symptoms, respectively (Amede et al., 2024).

The variations in prevalence rates of mental health disorders among refugees across the world may be influenced by the nature of the events experienced before, during, and after resettlement (e.g., experiencing life-threatening events, absence of social support, discrimination, language barriers, financial challenges, and cultural differences) in addition to methodological inconsistencies (e.g., different tools assessing PTSD, anxiety, or depression, either self-reported scales or clinical interviews, sampling techniques, and sample sizes).

### Factors Associated with PTSD, Anxiety, and Depression

Gender significantly influenced mental health outcomes among Palestinian refugees in Egypt. Females were more likely to experience PTSD and anxiety, while males showed higher levels of depression. These differences underscore the importance of gender-sensitive approaches in refugee mental health interventions. Previous studies supported our findings, showing that females are at a higher risk of PTSD (Altawil et al., 2023; Blackmore et al., 2020; Mahmood et al., 2019) and anxiety (Amede et al., 2024; El-Ghitany et al., 2024). This might be attributed to differences in the types of traumatic events experienced (e.g., sexual violence or becoming widows) as well as the influence of sex hormones on cognitive and behavioral functioning (Li & Graham, 2017; Riecher-Rössler, 2017). Another possible explanation is that females are typically more socially accepted to express their emotions openly compared to males (Chaplin, 2015).

While most studies reported that being female is highly associated with experiencing depression (Kuehner, 2017; Riecher-Rössler, 2017), our findings suggest a contrasting pattern. A similar finding was found elsewhere (Djukanović et al., 2015). However, a systematic review including 9,061 Syrian refugees found no relationship between sex and depression in four studies (Aysazci-Cakar et al., 2022). One possible explanation is that displacement may challenge traditional male gender role expectations, and men who perceive themselves as failing to fulfill these roles are more likely to experience psychological distress (Berke et al., 2022). Moreover, symptom presentation may differ by sex. Shi et al. (2021) found that women are more likely to report mild to moderate symptoms of depression, and men may underreport milder distress but are more likely to present with severe depressive symptoms. Together, these findings suggest that gender differences in depression among refugees may vary depending on cultural context, displacement experiences, and symptom severity, and should therefore be interpreted with caution.

Older age was associated with higher anxiety levels, reflecting the cumulative effect of prolonged exposure to stressors. Another possible explanation is that individuals who migrate at a younger age have more flexibility to adapt to the environment compared to older ages (Kemmak et al., 2021). Previous studies found that refugees older than 40 years had a significantly higher likelihood of experiencing mental health disorders (Albelbeisi et al., 2025; Amede et al., 2024).

Family history of mental diseases was significantly associated with depression, which was in alignment with previous studies (Colvin et al., 2014; Van Sprang et al., 2022). Almost all of the major psychiatric illnesses have a familial component in addition to various environmental factors (Ali et al., 2021). Van Sprang et al. (2022) reported that familial loading score (FLS), incorporating family- and disorder-specific characteristics (e.g., family size, prevalence of depression/anxiety), was associated with higher genetic vulnerability for major depression. Individuals with a higher depression FLS had more severe symptoms and an earlier age of onset.

Similar to our findings, Acarturk et al. (2021) and Lange et al. (2024) reported that the higher the socioeconomic status, the lower the PTSD among refugees. Additionally, a longitudinal study including 2,399 refugees found that poor financial status was consistently associated with mental ill-health for both male and female refugees over time during the resettlement process (Jiang et al., 2023). Post-migration challenges, such as employment difficulties and inadequate housing, were linked to higher mental health symptoms. Our finding implies that financial instability and obstacles in fulfilling basic daily living needs could contribute to persistent stress, raising uncertainty about the future and potentially triggering or exacerbating mental health problems (Jiang et al., 2023). Further, a systematic review highlights the critical role of housing as a social determinant of health for refugees (Rana et al., 2025).

Surprisingly, difficulty accessing medical services was associated with lower PTSD symptoms. This finding may be explained by avoidance symptoms in severe PTSD; consequently, the affected individuals would omit seeking medical help and thus not report access problems. Further, those reporting barriers may be more resilient and proactive in help-seeking, reflecting lower distress despite real obstacles. Cultural norms and stigma may also influence both symptom reporting and service use (Kirmayer et al., 2011).

Experiencing physical injury was significantly correlated with anxiety. In line with our findings, Tinghög et al. (2017) found that exposure to various types of trauma, both before and during migration, was associated with higher levels of anxiety, with physical violence identified as the strongest contributing factor. Further, a systematic review indicated that exposure to a greater number of traumatic events (e.g., living in a war-affected area; the experience of the death of someone close; experiencing a life-threatening accident, beating, or sexual abuse) was associated with poorer mental health outcomes (Aysazci-Cakar et al., 2022). Injuries do not only affect the body in the long term; they also amplify perceived threat and emotional distress, especially in vulnerable populations, like Palestinians who have experienced several wars that left physical and emotional scars.

Comorbidity among PTSD, anxiety, and depression was evident. Price et al. (2019) examined the comorbidity of PTSD, GAD, and depression with network analysis in trauma-exposed individuals. The results showed that the symptoms of depression and GAD were highly interrelated, while PTSD demonstrated notable heterogeneity. The comorbidity among these diagnoses is thought to arise from their overlap with negative effects (Price et al., 2019). Evidence also suggests that the co-occurrence of depressive and anxiety disorders is common, typically with anxiety disorders developing first. Over time, transitions are common between depressive and anxiety disorders. This comorbidity is associated with the worst functional, somatic, and psychiatric outcomes (Ter Meulen et al., 2021). Further, a study found that anxiety and depression shared genetic risk across the internalizing disorders (Hettema, 2008). Another study found that certain genes linked to being sensitive to trauma may be shared by both PTSD and Major Depressive Disorder (MDD) (Mundy et al., 2022). The comorbidity between PTSD and MDD also reflects overlapping symptoms in the two disorders (Flory & Yehuda, 2015). Additionally, a study indicated a medium effect size for the relationship between anxiety sensitivity and PTSD symptoms (Chiu et al., 2024).

Coping strategies significantly influenced psychological outcomes. According to the transactional model theory, the key variable between the individual and environment is the cognitive appraisal of the situation: first, the individual is exposed to a challenging event; then the external or internal demands of the situation are appraised according to the available resources for coping with those demands; and then a strategy of coping is initiated. These appraisals depend on individual differences and attitudes toward unexpected circumstances (Lazarus & Folkman, 1984). Literature indicates that engagement strategies correlate with an improved quality of life and higher life satisfaction, whereas disengagement strategies are associated with poorer psychological outcomes and lower life satisfaction (Bourguignon et al., 2020; Figueiredo & Petravičiūtė, 2025).

Interestingly, problem engagement showed a positive correlation with PTSD, suggesting that context and trauma type influence the adaptiveness of coping strategies. Although problem-focused coping is generally adaptive (Figueiredo & Petravičiūtė, 2025), our finding reflects the fact that there is no universal answer to the most effective coping strategy when facing life challenges, as the context plays an extremely important role (Stephenson & DeLongis, 2020). Additionally, the experience of traumatic events may influence how people employ coping strategies (Figueiredo & Petravičiūtė, 2025). Persistent cognitive engagement with trauma-related stressors may also intensify symptoms through rumination or heightened emotional arousal.

### Policy Recommendations

Mental health and psychosocial support (MHPSS) services should be tailored to refugees’ specific needs and integrated with programs addressing employment, housing, and education to mitigate stressors linked to mental health problems. Psychosocial care should also be incorporated into routine refugee aid programs to ensure sustainability and accessibility. Training of non-specialist healthcare workers and frontline staff in basic psychosocial skills is crucial to extend service coverage. Community-based interventions, such as peer-support groups and culturally sensitive outreach programs, are essential to strengthen social cohesion and resilience among refugees. Based on our findings, interventions should address gender- and age-specific vulnerabilities, trauma exposure, and coping strategies. Furthermore, digital and electronic MHPSS interventions, such as remote counseling and guided self-help programs, offer scalable, timely, and culturally adapted solutions in contexts of war and displacement (Ahmed & Heun, 2024).

### Limitations

This cross-sectional design limits the ability to establish temporal relationships between PTSD, anxiety, and depression. Future longitudinal studies are needed to better understand these associations. Further, the findings might be susceptible to social desirability due to the self-reported survey. Reliance on online surveys could introduce selection bias; however, 26.3% of participants were recruited through face-to-face interviews, which likely mitigated the under-representation of individuals without internet access. Nonetheless, the use of snowball sampling may have led to over-representation of individuals within similar social networks, and certain highly marginalized groups may still be under-represented. While the IES-6 is brief and practical for large-scale use, its cutoff score was validated in a non-war-affected population and should be interpreted with caution in this context. Future research should use a context-specific trauma scale. Further research is needed to explore additional factors influencing psychological distress among refugees and the displaced population, such as the availability and accessibility of mental health services and the wider basic services.

## Conclusion

This study highlighted the significant burden of the war on Gaza on the mental health of Palestinian refugees in Egypt and identified several factors influencing it (e.g., age, gender, financial status, and family history of mental illness, in addition to exposure to pre-migration and post-migration stressors).
